# Application of Electronic-Nose Technologies and VOC-Biomarkers for the Noninvasive Early Diagnosis of Gastrointestinal Diseases †

**DOI:** 10.3390/s18082613

**Published:** 2018-08-09

**Authors:** Alphus Dan Wilson

**Affiliations:** Pathology Department, Southern Hardwoods Laboratory, Center for Bottomland Hardwoods Research, Southern Research Station, USDA Forest Service, 432 Stoneville Road, Stoneville, MS 38776, USA; dwilson02@fs.fed.us; Tel.: +1-662-336-4809

**Keywords:** bacterial dysbiosis, early noninvasive diagnoses, electronic aroma detection, e-nose devices, GI-disease biomarkers, healthcare applications, metabolite profiles, point-of-care testing, volatile organic compounds

## Abstract

Conventional methods utilized for clinical diagnosis of gastrointestinal (GI) diseases have employed invasive medical procedures that cause stress, anxiety and pain to patients. These methods are often expensive, time-consuming, and require sophisticated chemical-analysis instruments and advanced modeling procedures to achieve diagnostic interpretations. This paper reviews recent applications of simpler, electronic-nose (e-nose) devices for the noninvasive early diagnosis of a wide range of GI diseases by collective analysis of headspace volatile organic compound (VOC)-metabolites from clinical samples to produce disease-specific aroma signatures (VOC profiles). A different “metabolomics” approach to GI disease diagnostics, involving identifications and quantifications of disease VOC-metabolites, are compared to the electronic-nose approach based on diagnostic costs, accuracy, advantages and disadvantages. The importance of changes in gut microbiome composition that result from disease are discussed relative to effects on disease detection. A new diagnostic approach, which combines the use of e-nose instruments for early rapid prophylactic disease-screenings with targeted identification of known disease biomarkers, is proposed to yield cheaper, quicker and more dependable diagnostic results. Some priority future research needs and coordination for bringing e-nose instruments into routine clinical practice are summarized.

## 1. Introduction

The procedures and methodologies used in clinical disease diagnostics are quickly evolving as new research reveals novel approaches, strategies and technologies to help speed up the prognostic cycle between clinical sample collection and final diagnostic determinations in Point-of-Care Testing (POCT) [1]. Major shifts in operating priorities of healthcare institutions, largely driven by higher medical costs and the need to improve on the delivery speed of medical solutions, are emerging as a worldwide trend to facilitate increases in efficiency and effectiveness of healthcare services. New diagnostic methods and procedures utilizing sensor arrays, with improved chemical-detection technologies and greater capabilities of chemical discriminations (particularly for distinguishing between complex gaseous mixtures of volatile metabolites derived from clinical samples), are being developed with the aim of improving both the speed and accuracy of disease diagnoses. For example, previous conventional methods used in the diagnosis of gastrointestinal (GI) diseases have involved the invasive or semi-invasive collection of clinical samples by colonoscopies, tissue biopsies, and microbial-swab cultures to assess causes of disease [2]. These intrusive, time-consuming and expensive methods are inappropriate for large-scale clinical disease-screening protocols and are being replaced by more cost-effective, noninvasive chemical-detection technologies and procedures that sense changes in volatile organic compound (VOC) metabolites. VOC metabolites are produced as a result of disease processes that alter the normal physiological and metabolic pathways occurring within disease-affected tissues of the GI tract. The increasing use of noninvasive, simpler and less expensive diagnostic methods for disease detection has become a significant new trend in many progressive diagnostic laboratories and clinics worldwide [3].

A variety of complex chemical-detection technologies, applying metabolomic approaches to disease diagnostics with complex instruments such as gas chromatography-mass spectroscopy (GC-MS), nuclear magnetic resonance (NMR) spectroscopy, selected ion flow tube-mass spectrometry (SIFT-MS), ion molecule reaction-mass spectrometry (IMR-MS), secondary electrospray ionization-mass spectrometry (SESI-MS), proton transfer reaction-mass spectrometry (PTR-MS), and field asymmetric ion mobility spectroscopy (FAIMS), recently have been used to identify disease-associated VOC-metabolites [4,5,6,7,8]. By contrast, the cheaper and more portable diagnostic devices, such as electronic-nose (e-nose) instruments, are capable of detecting and discriminating between complex mixtures of disease-associated volatile metabolites without identifying individual chemical species. E-noses are gas-sensing systems that generally contain cross-reactive multi-sensor arrays capable of discriminating between sensor-response patterns of VOCs by using pattern-recognition algorithms for aroma classification [9]. The simpler e-nose approach to disease diagnostics offers a potential means for noninvasive early diagnosis of GI-tract diseases with greatly accelerated prognostics, allowing earlier more effective treatments, more rapid patient recovery, and shorter less-expensive stays for hospital care [1,10,11,12].

A wide range of electronic-nose instruments, based on different operating technologies and chemical-detection sensor types, have been used in clinical disease diagnostic applications. The most common types of e-nose devices used in clinical practice include sensor arrays with surface acoustic wave (SAW), quartz crystal microbalance, metal oxide semiconductors (MOS), carbon black polymer composite (CBPC), conducting polymers (CP), and carbon nanofiber (CNF) sensor types as representatives of electronic aroma detection (EAD) technologies [13,14]. Design details of sensor characteristics such as sensitivity, VOC-sensitive components, detection mechanisms, response times, advantages and disadvantages of each e-nose type were summarized previously [8]. E-nose technologies have been tested extensively for numerous broad applications in the biomedical, forensic, chemical and pharmaceutical industries [14,15,16,17,18]. A diversity of commercial applications for e-nose devices also have been developed within the fields of agriculture, bioterrorism prevention, cosmetics, environmental protection and monitoring, food and beverage, fragrance, manufacturing, military, regulatory, transportation security and wood-products industries [19,20,21,22].

This review summarizes recent applications of electronic-nose devices for the noninvasive early detection of infectious diseases caused by microbial pathogens and some noninfectious diseases common to the human intestine. Disease theory relating to the diagnosis of GI-tract diseases having both biotic and abiotic causes is discussed. Two relatively new approaches to GI-disease diagnostics, including the use of metabolomic methods to identify disease VOC-biomarker metabolites and simpler electronic-nose devices, are compared and evaluated based on diagnostic costs, accuracy, advantages and disadvantages. In addition, new concepts are introduced for the potential use of new dual-technology approaches to disease diagnostics which take advantage of the rapid early prophylactic disease-screening capabilities of e-nose instruments that could be used in combination with the targeted VOC biomarker-detection capabilities of metabolomic technologies. This new approach could potentially yield more rapid and dependable diagnostic results. A wide range of GI-diseases have been detected using both metabolomic and e-nose instruments and methods included in this review. A complete list of abbreviations for all diseases, analytical instruments, sensor types, clinical methods, and VOC-metabolite (analyte) categories are provided in Appendix A.

## 2. Gastrointestinal Disease-Detection Methods

Numerous studies have demonstrated the well-established efficacy of utilizing VOC profiles of headspace metabolites derived from diseased tissues for clinical diagnostics by two completely separate approaches: electronic-nose and metabolomic analyses that utilize different sensing technologies [15,20,23,24,25]. The main difference between these two diagnostic approaches lies in the specificity of chemical detections of components within clinical samples. Metabolomic approaches to disease diagnostics attempt to detect the occurrence, identity, and changes in quantities of VOCs and sometimes nonvolatile chemical species present in clinical samples. By contrast, e-nose instruments aim to detect collective differences in the chemical composition of all VOC-metabolites (including complex mixtures of chemical biomarkers) present in clinical samples. Each approach provides different types of information useful for diagnostic determinations.

A comparison of the efficacy and practicality of metabolomic verses e-nose diagnostic methods of VOC-profile analysis, based on capabilities, advantages, disadvantages and performance, provides a means for assessing the suitability of each technology in meeting the needs of modern clinical requirements for disease-detection applications. Metabolomic analytical methods have numerous disadvantages that preclude these technologies from meeting the essential criteria to achieve effective application and performance for daily clinical use. These complex analytical instruments usually are expensive to purchase, operate and maintain. They require labor intensive operations by highly trained and skilled operating personnel. Following analytical runs, data analysis takes considerable time due to the required use of advanced statistical analyses that often apply complex molecular models before data interpretations are possible. Consequently, metabolomics methods do not allow for adequate sample throughput in a timely manner for real-time diagnostic applications. Such systems are usually not portable for use in clinical examination rooms, patient care rooms or for emergency field situations.

Electronic nose devices are much simpler compared to metabolomics instruments and offer considerably more advantages that make them more suitable for routine clinical use. E-noses usually are inexpensive to purchase, operate and maintain. They have the capability of real-time, high-throughput analysis and produce data outputs that are relatively easy to interpret using well-designed utility software. E-nose analytical results generally are based on pattern recognition algorithms using software that compares VOC-profiles of analytes (headspace volatiles of clinical samples) to profiles of known samples (e.g., healthy vs. diseased patients) in predeveloped diagnostic libraries. The main limitation of e-nose devices is their inability to determine the identity and concentration of individual VOCs, except for combined-technology instruments. Differences in the various types of e-nose instruments that have different operating principals precludes data comparisons of inter-device results. Furthermore, the reproducibility of e-nose results can be affected by sensor drift over time, reducing instrument reliability. Some key advantages and disadvantages of metabolomic verses electronic-nose analytical methods are summarized in Table 1.

Early disease diagnosis is of paramount importance in order to achieve rapid and effective treatments for diseases to greatly improve prognoses for patient recovery [8,14,25]. Consequently, diagnostic methods must provide quick results, particularly for fast-acting diseases and systemic infections (sepsis) by morbific pathogens that cause rapid death of patients without prompt effective treatments. In these situations, any time delay in diagnoses and applications of immediate curative treatments may result in irreversible damage, greatly reducing any chances of patient recovery. Conventional methods and metabolomic technologies usually do not produce useable diagnostic information fast enough to provide effective healthcare services to patients.

Some difficulties have been experienced with metabolomic approaches due to the inability of obtaining reliable disease diagnoses based solely on relatively few disease biomarkers. Other problems include the small number of known biomarkers for specific diseases, the large variability in VOC metabolites caused by external factors not related to the disease condition, and the relatively small number (<100) of metabolites detected so far with this approach [3].

Analysis of VOC metabolites emitted from fecal samples have shown significant changes in VOC profiles that have facilitated determinations of disease etiology and identification of potential disease biomarkers associated with specific GI diseases [23]. Changes in VOC profiles of the GI-tract normally result from alterations in host physiology due to disease processes (pathogenesis) that often cause changes in gut microbiota composition as a consequence of changes in GI-growth conditions for microbes in the gut of diseased patients compared with healthy patients. Pathogenic microbes that cause biotic diseases usually produce changes in the VOC composition of headspace GI-gas mixtures due to the release of unique types of pathway-specific metabolites from fecal samples extracted from a diseased gut. Complete analyses of VOC profiles provide a clearer understanding of the mechanisms (pathophysiology) and origin of disease resulting from changes in host metabolic pathways [3]. Exhaled breath VOCs associated with inflammatory bowel disease probably arise from microbe-associated gases originating in the gut that diffuse into the bloodstream and are released into lung alveoli [26]. Certain VOCs highly associated and correlated with the presence of specific diseases and metabolic pathways may serve as effective chemical biomarkers of disease.

### 2.1. Biomarker Metabolites

Continuous advances in modern healthcare toward personalized or precision medicine, known more widely as stratified or P4 (predictive, preventive, personalized, and participatory) medicine have resulted in the development of new chemical methods of disease surveillance [27,28]. The discovery of disease-related chemical biomarkers has emerged as a key tool that has moved this process forward by providing objectively-measurable indicators (chemical signatures) to indicate the presence of normal or pathogenic metabolic processes or physiological states within the body [29]. In this way, disease biomarkers have become significant contributors to disease diagnosis, patient therapy, clinical trials and personalized medicine [30,31,32]. Unfortunately, slow progress in disease biomarker discovery and application development has thwarted the widespread use and implementation of biomarkers in clinical practice.

Chemical biomarkers of disease consist primarily of two major categories of organic compounds including nonvolatile proteins, (such as cytokines, chemokines, cell surface markers and acute phase reactants), and volatile metabolites of many chemical classes which are products of cellular metabolic processes, usually derived from either host or microbial (often disease-causing or pathogenic) origin [33,34,35,36,37]. Additional disease biomarker candidates are being investigated and discovered through genomics, proteomics, nucleic acid-based molecular techniques and metabolomics [38].

Unique disease-specific chemical biomarkers may be identified to detect and diagnose disease states within the body, predict patient physiological responses to chemical therapies, monitor disease progress and regressions or recurrences [39]. Chemical biomarkers identified from clinical samples include body fluids (such as blood, urine, serum, and saliva), exhaled breath, feces, sweat and excretions from various tissues samples. Detection of disease biomarkers is a complicated process because many factors affect the production, occurrence and concentration of VOC metabolites within clinical samples. Disease processes and mechanisms vary for different disease types and cause different consequential changes in host metabolism and physiology that may be analytically detected and monitored. The location of diseased tissues within the body affect the characteristics of metabolic profiles. VOC profiles of diseased individuals may be compared to normal VOCs present in healthy individuals. Many recent metabolomics studies of human diseases have involved analytical methods to monitor VOC metabolites of diseased verses healthy patients to identify possible biomarker metabolites as chemical indicators of specific diseases. Diverse VOC-metabolites found useful for the noninvasive early diagnosis of some important diseases are presented in Table 2.

Progress in the discovery of disease biomarkers in the fields of veterinary and aquaculture sciences has facilitated the diagnosis of animal diseases and provided clues to identifying possible biomarkers of similar diseases in humans. Disease-related biomarkers recently have been identified for catfish, livestock, and wildlife diseases which potentially may be further developed for the noninvasive early detection of these diseases in both aquatic and terrestrial environments using e-nose devices [41,48,49,50,51,52,53,54]. The discovery of two major VOC-biomarker secondary metabolites, geosmin (1,10-trans-dimethyl-trans-(9)-decanol) and 2-methylisoborneol (MIB), associated with systemic infection of live catfish by aquatic actinobacteria, were determined to be the main VOCs present or absent in the headspace of raw skinless catfish flesh. These factors were responsible for effective CP e-nose discriminations between “off-flavor” and “good-flavor” meat fillets determined by principal component analysis (PCA) [48,55,56].

Volatile biomarker metabolites detected in clinical samples are derived from different classes of organic compounds that are indicative of the metabolic pathways (either of host, pathogen, disease, or endophytic microbiome origin) from which they were derived. The presence of abnormal disease-associated metabolites within clinical samples often provide specific informative clues to the nature and types of metabolic pathways affected by the disease. This information often relates to the particular mechanism of a disease, determined by the processes by which a causal agent induces pathogenesis, and the resulting specific effects of disease-associated processes in altering or diverting normal (healthy) metabolic process within diseased tissues of the host. Microbial pathogens often utilize different types and combinations of pathogenic determinants (including enzymes, proteinaceous and organic toxins, polysaccharides, and genetic-regulatory factors) to initiate disease within hosts. The particular types of pathogenic determinants involved in disease development largely determine the mechanisms of disease and host metabolic processes and pathways affected by pathogenesis. Therefore, metabolomic identification and categorization of volatile biomarker metabolites (by host pathways affected) may provide useful clues of disease mechanisms operating in affected tissues and types of microbial pathogens and pathogenic determinants involved.

Previous analyses of the VOC-portion (headspace) of the metabolome from excreted materials (blood, urine, feces and breath) has proven to be a promising screening tool for the diagnosis of several cancers, including colorectal cancer (CRC), according to a recent review [57]. The VOC-signature in the form of a smellprint (similar to a fingerprint) serves as a reference-gauge that reflects the overall health status of a patient which is modified by cancers in the GI-tract. This review concluded that technical difficulties, such as finding suitable and consistent disease biomarkers that are produced despite numerous variability factors, still have limited the widespread use of VOC-analyses in the clinical setting. However, this approach has been applied successfully with many examples indicating good reliability of the metabolomic approach in detecting CRC, via identification of different VOC-signature patterns, despite several limitations due to variability in number of patients included in each study, different analytical platforms and biological materials used, and different VOC-biomarkers identified.

The current diagnostic clinical processes followed for diagnoses of pediatric GI disorders still usually includes invasive tests that are painful, especially to young patients. This problem has pointed to the need for novel, noninvasive disease-specific biomarkers for GI tract diseases. The wide diversity of VOCs, originating from patho-physiological metabolic processes in the human body, are excreted as waste products within bodily excrements, providing a unique source of chemical information offering great potential for monitoring disease activity through the detection of noninvasive diagnostic biomarkers of GI diseases. VOC analysis has been studied in children and infants with a variety of GI diseases, including inflammatory bowel disease, liver diseases, irritable bowel syndrome, necrotizing enterocolitis and infectious diarrhea [58]. Most studies have shown that diseased patients may be discriminated from healthy patients based on differences in their VOC profiles, although the application of VOC-analysis in clinical practice is somewhat limited by procedural and technical challenges including methodological, biological and analytical problems. The VOC analytical techniques requiring further refinement before VOC diagnostics is implemented on a widespread basis in standardized clinical practice were reviewed previously [58].

Endometriosis metabolomics was investigated using a similar approach to explore diagnostic disease-specific biomarkers by analyzing changes in metabolic profiles [44]. Twelve metabolites were identified in this study as potential endometriosis-associated biomarkers within the eutopic endometrium metabolomic profile. Profiles of endometriosis patients were characterized by significant increases in concentrations of hypoxanthine, L-arginine, L-tyrosine, leucine, lysine, inosine, omega-3 arachidonic acid, guanosine, xanthosine, lysophosphatidylethanolamine and asparagine. This study provided the first comprehensive analysis of metabolic changes in the eutopic endometrium for endometriosis at early stages. The potential biomarkers for early diagnosis of endometriosis were identified as metabolites of the purine, amino acid and arachidonic acid metabolic pathways. These potential endometriosis biomarkers were found to be applicable for semi-invasive (laparoscopically confirmed endometriosis at stages I–II) diagnosis of endometriosis at minimal or mild stages in clinical practice.

A new instrument, called a GC-Olfactometry (GC/O) device, recently was investigated as a means of targeting the identification and analysis of potential GI-cancer biomarker metabolites in urine samples [59]. This pilot study utilized Headspace Solid Phase Microextraction (HS-SPME-GC/MS) in combination with GC/O-analysis to compare the headspace VOC composition of urine samples obtained from gastrointestinal cancer patients and healthy controls. They found 91 key odor-active compounds in urine samples of cancer patients and significant differences in 11 VOCs were discovered that discriminated between healthy and diseased patients. Seven compounds were identified: Thiophene, 2-methoxythiophene, dimethyl disulphide, 3-methyl-2-pentanone, 4-(or 5-) methyl-3-hexanone, 4-ethyl guaiacol and phenylacetic acid. The main conclusion of the study was that targeted GC/O had greater potential for detecting cancer-related biomarkers that were not detectable using untargeted GC/MS approach. The GC/O instrument may provide a means of improving the identification of cancer-associated compounds as potential biomarkers in clinical practice to further support early cancer diagnosis.

### 2.2. New Metabolomic Disease-Detection Approaches

Metabolomic approaches to disease diagnosis attempt to detect changes in the types and quantities of specific volatile organic metabolites (VOMs) produced by disease processes. These methods require extensive knowledge and skills in the use of sophisticated chemical-analysis instruments as well as complicated chemical-modeling methods and statistics software. The standard method of metabolomics analyses normally involves GC-MS analyses often in combination with NMR spectroscopy for identification of the types and quantities of VOMs present in the headspace of clinical samples. More recently, SIFT-MS and FAIMS-analysis are additional tools used in the detection and identification of candidate biomarkers that may be useful to detect and diagnose diseases in the GI tract.

Fecal samples of 140 patients with chronic irritable bowel syndrome (IBS), analyzed by GC-MS with univariate statistical analysis, revealed 240 VOMs of which 44 compounds were identified that could be used to discriminate between patients with diarrhea-predominant Crohn’s disease and ulcerative colitis forms of IBS [23]. Inflammatory bowel disease (IBD) was investigated in a similar study, using partial least-squares-discriminate analysis of GC-MS data, which showed effective discrimination between patients with Crohn’s disease and healthy controls based on the greater or lesser abundance of eight specific VOM-biomarkers from different chemical classes [60]. In this study, three unique biomarkers (1-octene, 1-decene, and (E)-2-nonene) were identified in the breathprints of children with IBD, determined by metabolomic analysis of breath VOCs using linear discriminant principle component analyses (LD-PCA) with SIFT-MS data [61].

Profiling headspace VOCs using portable FAIMS technology may have significant advantages over conventional SIFT-MS chemical analyses. FAIMS technology, now available as a small, portable POCT breath-analysis device that uses air as the carrier gas and provides real-time discriminations of VOC profiles. A comparison study has indicated that FAIM operates at a fraction of the cost (10%–20%) of SIFT-MS, but SIFT-MS outperforms the diagnostic power of FAIMS [62]. FAIMS-analysis of headspace urinary VOC signatures using Fisher discriminant analysis recently was used in the detection of colorectal cancer [63]. The FAIMS method of VOC analysis also has been used to differentiate diagnoses of coeliac disease from IBD by analysis of headspace urinary VOCs along with the discovery of 1,3,5,7-cyclooctatetraene as the single VOC biomarker of CD that was absent in urine from IBD patients [64].

### 2.3. Biomarkers of Microbial GI-Tract Diseases

A variety of different categories and classes of volatile chemical biomarkers have been identified in the gut of diseased patients that may be used to detect and correlate with the occurrence of various types of disease states in the intestines and colon. Some of the most significant identified categories of disease-biomarkers associated with GI diseases, based on the biochemical origins of VOCs produced, include predisposition, disease (pathogenesis), pathogen and gut-microflora biomarkers [3]. Each of these categories of disease biomarkers, produced by different sources and mechanisms (as a consequence of disease), provide different information about the source, chemical nature, and effects of the disease on host-targeted pathways affected and changes in gut chemistry as a consequence of disease. Disease-predisposition biomarker metabolites originate from behavioral, genetic, or environmental factors that previously predisposed patients to certain disease types prior to disease development. Thus, predisposition VOC-biomarkers are useful for screening patients for susceptibility to specific diseases much like genetic markers. Disease-related biomarkers result from physiological alterations in normal host metabolic pathways as a direct consequence of pathogenesis. Pathogen biomarkers are VOC-metabolites that originate from the metabolic pathways of pathogenic microbes (pathogens themselves) that cause biotic infectious diseases. Gut-microflora biomarkers originate from new microbes (often different from normal resident bacteria) that arise in the gut due to significant changes in the chemical environment (nutritional or substrate composition) resulting from disease effects on intestinal tissues. The compositional alteration of resident commensal microbiome communities in healthy individuals due to disease has been referred to as bacterial dysbiosis [65]. These unique microbial and metabolic shifts that occur within the gut, preceding disease onset and symptom development, provide unique opportunities for early noninvasive disease detection.

The chemical classes of disease biomarkers provide useful insights into the specific host metabolic pathways affected by disease processes. By this approach, the appearance or accumulation of particular intermediates and derivatives of common metabolites (within specific pathways) provide indications of both the mechanisms of disease (how disease processes are initiated by pathogenic determinants), and the precise means by which pathogenesis affects metabolic pathways that lead to useful disease biomarker production. For example, disease-associated alterations in the tricarboxylic acid (TCA) or Kreb cycle pathway tend to produce greater or lesser quantities of organic acids or carboxylic-acid derivatives [3]. A summary of some of the most important biomarker metabolites discovered in association with specific types or categories of GI-tract diseases, listed with the causal agents, metabolomic-type analytical methods used for identification, source and type of clinical samples analyzed, and key VOCs identified in each study is presented in Table 3. Notes are provided to indicate the chemical classes of each biomarker and whether the presence or absence of the biomarker was established to indicate the likely occurrence or absence of the disease relative to compound abundance and presence in healthy control patients.

Gut microbiota commensals play a very substantial role in maintaining human immunity (immune system regulation), metabolic homeostasis, nutrient digestion and uptake, and in modulating immune-cell differentiation, maintaining intestinal barrier integrity, and preventing epithelial cell injury [66,67]. Microbial commensals also provide direct controls of GI-tract pathogens by direct interactions including production of bacteriocins, chemically altering the GI environment, competitive inhibition and boosting host resistance mechanisms [66,68,69].

Disease VOC-biomarker detection via metabolomic methods, used either individually or in combination with various e-nose technologies, has been shown to be a powerful and complementary tool for effective disease diagnosis [26]. Significant differences in VOC-metabolite patterns derived from GC-MS headspace analyses of stool samples showed that the main causes of infectious diarrhea in hospitals could be discriminated based on unique biomarkers associated with different infectious etiologic agents [42]. *Clostridium difficile* produced furan biomarkers (without indole functional groups) whereas rotaviruses induced the production of an ethyl dodecanoate (ED) biomarker, but enteric viruses induced the production of ammonia without the ED-biomarker, and *Campylobacter* species did not produce terpenes and simple hydrocarbons.

Two additional studies showed that detection of three specific exhaled VOCs (including 1-octene, 1-decene and (E)-2-nonene) found in breathprints could be used to distinguish between pediatric patients with IBD and healthy controls [61,81]. All three of these potential IBD-related biomarkers are relatively short-chain alkane hydrocarbons.

Giardiasis, caused by the flagellated intestinal protozoan *Giardia duodenalis*, is a common intestinal infection of people who drink unfiltered or unsterilized water, particularly from non-municipal water supplies. The life cycle of this parasite alternates between a swimming, water-borne trophozoite and an infective, resistant cyst. The most common methods available for laboratory diagnosis of Giardia include microscopic identification of the parasitic trophozoite and cyst stages, immunodiagnosis and PCR. Fortunately, Giardia has unique metabolic pathways, resulting from its lack of mitochondria, making it an ideal target for VOC-profiling in giardiasis diagnosis. Bond et al. [71] analyzed fecal headspace gases by GC-MS and found that patients with giardiasis predominantly had VOCs including esters, organic acids, alcohols, ketones and aldehydes, minor components of benzoids, alkanes, heterocyclic compounds and a few amides, alicyclic and ether compounds that were not found in healthy patients.

The unexplained possible relationship between biomarker metabolites found in association with stomach ulcer and those released by cancerous stomach tissues was recently investigated. VOC-profile analysis comparisons showed overlaps of eight VOCs between cultured *Helicobacter pylori* and volatile compounds released from stomach cancer tissues [82]. Seven cancer biomarkers were identified including: Carbon disulfide, 1-propanol, 2-propanol, 2-butanone, 4-methylheptane, 4-methyloctane and 2-ethyl-1-hexanol. The detection of carbon disulfide and 1-propanol VOCs found in common between *H. pylori* and cancerous tissues suggested that the *H. pylori* bacterium may have been present in conjugation with cancerous tissue to explain the commonality.

Gut microbiome studies have shown significant roles of different types of GI-microbiota in protecting against (promoting health) or exacerbating human diseases caused by gastrointestinal disorders, metabolic diseases and inflammatory diseases [83]. Intestinal microbiota promote health or facilitate disease due to their metabolic functions through catabolic fermentations, short-chain fatty acid production and vitamin synthesis [84,85]. Fecal-metabolite profiling has been used in combination with microbiota profile analysis to discover potentially useful biomarkers for diagnosing various non-infectious disorders and GI-tract diseases, such as CRC, chronic gastrointestinal disease, celiac disease, nonalcoholic fatty liver disease and necrotizing enterocolitis (NEC) [80,86,87,88]. The presence of *Phascolarctobacterium* and *Acidiminobacter* bacteria in fecal samples, along with the presence of the amino acids phenylalanine and glutamate, has been correlated with the intestines of healthy patients, whereas the amino acids serine and threonine were found in greater abundance in the fecal samples of CRC patients [89]. Higher concentrations of esters, indole and short-chain alcohols derived from fecal bacteria in Crohn’s disease patients, compared with healthy controls and patients with ulcerative colitis, provide another example of how VOCs, produced by changes in gut bacterial microbiome in response to disease, can be used for disease diagnosis [90]. Fecal microbiota VOC-profile analysis using metabolomic studies (qualitative and quantitative analysis of fecal metabolites) often provide data that can be used for developing rapid and sensitive diagnostic methods for GI-diseases [82,91].

Probert et al. [92] analyzed fecal samples from 30 asymptomatic individuals and identified 297 VOCs, consisting primarily of organic acids, alcohols and esters and forty-four compounds that were common to 80% of these samples. By contrast, VOCs identified in the stool of patients with *Clostridium difficile*, *Campylobacter jejuni* and ulcerative colitis strongly suggested specific changes in the pattern of VOCs in patients with GI disease that could be utilized for diagnoses in clinical settings [93].

De Lacy Costello et al. [94] conducted analyses of fecal VOCs from neonates and found fewer VOCs, including lower frequency of nitrogen compounds and no sulfides compared with healthy adults, reflecting the simplicity of neonatal gut microflora. Additional work on gut VOCs of neonates with NEC showed that infants had fewer esters than healthy individuals. This change occurred before NEC was diagnosed by clinicians, indicating a possible means of early NEC detection.

The pathogenesis mechanisms of NEC and late-onset sepsis (LOS) in preterm infants have yet to be elucidated, but there is emerging evidence that gut microbiota plays a key role in the pathophysiology of these diseases [66]. For both NEC and LOS, preclinical alterations in gut microbiota composition and fecal VOCs provided evidence that microbiome metabolism of collective gut microbes is important in determining fecal VOC composition changes as a result of these two diseases. Although a NEC- and sepsis-specific microbial metabolic signature has not yet been discovered, further investigations of disease-specific VOCs and relationships with microbiota composition may increase understanding of ways for developing an accurate screening tool for early detection of these diseases in order to provide timely targeted treatments for preterm infants at increased risk for NEC and sepsis. Additional studies associated with altered gut microbiota composition in preterm infants with LOS, up to several days before clinical onset of sepsis, suggested that analysis of fecal VOCs may serve to help identify noninvasive biomarkers to predict LOS at a preclinical stage since VOCs reflect the composition and activity of intestinal microbial communities [95]. The common occurrence of bacterial dysbiosis offers great promise for GI-disease detection.

Stewart et al. [79] obtained preliminary metabolomic data to suggest that gut development and protection in preterm infants is associated with increased prebiotic oligosaccharides (e.g., raffinose) and the growth of beneficial (protective) bacteria, such as *Bifidobacterium* species. Bifidobacteria dominance association with control (healthy) infants indicate that this microbe may directly protect the gut against disease, while metabolites produced by these bacteria may serve as an indicator for protection against gut epithelial translocation. Multi-omic and sPLS correlation analysis shows distinct correlations between significant metabolites and dominant gut bacterial genera present. The presence of *Bifidobacterium* and *Streptococcus* species showed strong positive correlations with a range of metabolites that were significantly increased in healthy (control) infants, including raffinose, 18-hydroxycortisol, 18-oxocortisol, acetic acid, and L-alpha-acetyl-N-normethadol [79].

## 3. GI-Disease Types and E-Nose Methods for Detection

The most important GI-tract diseases detected with e-nose technologies include colorectal cancer, inflammatory bowel disease including Crohn’s disease and ulcerative colitis, irritable bowel syndrome, infectious diarrhea, celiac disease, necrotizing enterocolitis and cholera [24]. All of these GI diseases have been diagnosed with e-noses based on the presence of specific complex mixtures of abnormal headspace VOCs, released from feces in diseased patients, that produce disease-specific sensor output patterns (from an e-nose sensor array) in response to complex VOC mixtures and identified using pattern-recognition algorithms and disease-specific reference libraries.

### 3.1. Clinical Sample Types for GI E-Nose Analyses

Current clinical methods used for e-nose detection of GI-tract diseases are determined by the location and etiology of disease, mechanism of pathogenesis, the classes of VOC-metabolites produced as a result of disease within affected tissues, and the mobility of disease-associated volatile metabolites, including translocation within the body and release from excretory systems (pulmonary, urinary, and gastrointestinal) [3,15]. Thus, the type of clinical samples collected for e-nose disease detection and diagnoses are disease-specific and relate to the precise locations where chemical indicators (such as disease biomarkers) concentrate and may be detected for diagnostic evaluations.

Clinical samples utilized to diagnose various types of GI diseases based on headspace gases include human breath, urine, blood serum and fecal (stool) sample types. Analysis of headspace VOCs released from fecal samples reflect the overall metabolic state of an individual and the presence of disease states within the body. A wide variety of factors affect intestinal metabolism including diet, medication and drug use, ingestion of probiotics and antibiotics, types of gastrointestinal resident microbes (GIRMs) present (which comprise the GI-microbiome) and presence of disease within the body [3,23]. Significant alterations in host metabolism (physiology) caused by GI-tract infections, inflammation and related disease states result in changes in GIRM-composition, altering the complex mixture of VOC gases released from fecal samples. Disease-associated changes in GI-microbiome often amplify the changes in headspace volatiles induced by disease processes. Modern chemical-detection devices sense changes and differences in VOC metabolite signatures (profiles) in diseased patients, compared with those of normal healthy individuals, to diagnose many different types of GI-tract diseases. Detection of specific known VOC chemical biomarkers by metabolomics methods may provide confirmation of disease diagnoses determined from unique fecal VOC smellprint patterns.

The diagnostic accuracy of VOC profiles utilized for diagnostic determinations are largely determined by the quality of clinical samples analyzed. Sample collection methods and storage conditions prior to analysis may significantly affect the stability and reproducibility of gas vapor emissions from various sample types, depending on the disease and nature of the VOCs analyzed. Some clinical sample types may degrade while in storage. Thus, sample shelf-life must be considered and determined when samples are stored for long periods because the quantities and diversities of total gas VOC emissions may be reduced over time. Specific examples of the importance and effects of sample quality and type on diagnostic accuracy are given in Section 3.4, which describes e-nose applications and methods for specific diseases.

### 3.2. Importance of QA/QC in E-Nose Disease Detections

The reliability of e-nose analytical information is highly dependent on maintaining effective quality assurance/quality control (QA/QC) procedures during instrument operations. Sensor drift due to sensor degradation may significantly affect the precision of e-nose analyses from different experimental sessions, potentially causing inconsistent adverse effects on long-term instrumental performance and training if not adequately addressed. Many modern e-noses have built-in software features that provide warnings (via auto-tests that check for sensor drift and any operating parameters outside of run settings), prompting instrument recalibration procedures when necessary to automatically fix QA/QC problems and normalize sensor responses after instrumental recalibration. This recalibration process largely compensates for any systematic errors in sensor-array output that may affect instrument performance.

Sensor arrays in certain e-nose types also may be highly susceptible to inactivation by specific classes of VOCs, particularly sulfur-containing compounds, organic acids and highly polar pesticides [20]. Some e-nose sensor types inherently are more stable and less susceptible to aging or inactivation by poisoning than others. MOS e-nose sensors tend to have much longer life and are considerably less susceptible to sensor inactivation by chemical agents than CP sensors. Consequently, e-nose instrument stability may be maintained through recalibrations that provide effective analyte discriminations for many years. For sensor arrays sensitive to inactivation, sensors that become corrupted can be turned off if not necessary for effective discriminations. Otherwise, the entire sensor array may be replaced if sensor degradation causes instrument performance to fall below operational standards.

The performance of most e-nose instrument types is strongly affected by variations in the relative humidity of the carrier gas (air) and moisture in the headspace sample. Water vapor often is perceived by e-nose sensors much like VOCs and can affect the adsorption of VOCs to sensor surfaces due to dynamic equilibrium effects [19,20]. Water molecules in moist sampling air competitively adsorb to sensor surfaces and may cause very significant impacts on e-nose sensor responses. The presence of water vapor in the sample also may result in preferential detection of hydrophilic over hydrophobic VOCs. To prevent these problems, reference air must be maintained at low relative humidity (usually <5% moisture content) to minimize moisture effects on sensor responses. One notable exception is CPBC e-nose sensors that are not significantly affected by moisture. Thus, CBPC e-noses may be used to discriminate between complex headspace VOC analyte mixtures with high moisture content. The routine treatment of reference air with a desiccant or built-in relative humidity control device is necessary to minimize moisture effects. The lack of control of relative humidity in clinical samples may significantly reduce instrument discrimination power.

The quality and diversity of known diagnostic samples used for e-nose training and for setting up diagnostic reference libraries, such as application-specific reference databases (ASRDs), is critical to achieving effective sample discriminations for diagnostic applications. Known diagnostic samples used for e-nose training should be representative of the full range of possible sample variation for each particular disease type and include sufficient replications of reference samples within the geographic region (human population) from which the samples were taken to achieve high power in statistical discriminations [8,19]. Consequently, for e-nose library reference databases (of VOC profiles) to be representative, they should be created from known diagnostic samples taken from the indigenous human population of the geographical region from which most clinical patients are living in order to eliminate the effects of geographical, racial or cultural variation in diagnostic samples.

The sampling methods and sample-discrimination parameters used in e-nose clinical determinations are equally important factors in achieving reliable diagnostic results [16,19]. The specificity of criteria used for sample discrimination is largely determined during e-nose training sessions. Known clinical sample types (for particular diseases) are used in creating diagnostic reference libraries and the level of discrimination specified for particular types of diagnostic sample determinations are usually controlled by training duration. If the instrument is either over-trained (sample-discrimination criteria specified is too high) or under-trained (sample-discrimination criteria specified is too low), this will result in ineffective and inaccurate sample discriminations with both false positive and false negative results as possible outcomes. The level of sample-discrimination parameters required to achieve effective e-nose discriminations must be determined empirically for each disease and instrument type using high quality known reference samples.

Individual portable e-nose instruments generally perform much more effectively when dedicated to the detection and diagnosis of a particular disease or a small cluster of closely related diseases often having clinical samples with very similar VOC composition. Optimizing sample discrimination parameters to most efficiently discriminate specific clinical sample types usually provide superior results. Part of the reason for this improved e-nose performance when the e-nose instrument is tailored for detection of specific diseases lies in the effective use of disease-specific sample discrimination parameters and the potential use of ASRDs that minimize false discriminations.

### 3.3. Electronic-Nose Instruments for GI-Disease Detections

Electronic-nose devices are instruments capable of detecting complex mixtures of VOC-biomarker metabolites that occur within headspace volatiles derived from clinical samples. Each individual sensor within the sensor array of an e-nose responds simultaneously and collectively to all of the chemical compounds present (in various concentrations) within the gaseous analyte mixture. Combined together, the responses of all of the sensors within the e-nose sensor array are translated by a transducer and converted to a digital signal sensory output in the form of a collective sensor-response pattern that may be used to identify a very specific type (mixture) as well as the origin (source) of VOC-metabolites derived from healthy or diseased tissues. The recognition process of a sample type usually is achieved through the use of pattern-recognition algorithms and specific statistical software in combination with machine neural net learning linked to smellprint reference library databases of known sample type signatures previously recorded from known healthy and diseased tissue samples from known host species.

E-nose devices useful for discriminations of complex VOC-metabolite mixtures found in headspace volatiles of GI diagnostic samples have been reviewed previously [96]. Different e-nose sensor-array types have varying advantages that determine their potential range of clinical applications and sensitivities to particular classes of chemical analytes associated with disease detection [3,8,15]. One notable limitation of applying e-noses to medical diagnostics is the difficulty of using a particular e-nose unit, trained for detecting one particular disease, to be reprogrammed and trained for detection of a different disease [97]. Nevertheless, the versatility, high adaptability for specialized applications and relative simplicity of e-nose devices makes their development and modification for many potential diagnostic applications quite attractive and compelling for the field of medicine [15,20].

### 3.4. Recent E-Nose GI-Disease Detection Applications

The arrival of new experimental e-nose devices with novel early disease-detection methods and sensor types for VOC profiling are promising additional tools for achieving more rapid and effective clinical diagnostics. Some of the most recent examples of e-nose devices being used for detection of GI-tract diseases are summarized in Table 4. These devices represent different EAD technologies with different operating principals for detection and numbers of detectors in the sensor arrays.

The older technology e-nose types still in use for diagnosis of GI diseases include metal oxide semiconductor, carbon black polymer composite, electrochemical (EC) and gold nanoparticles-type electronic noses. The newer more loosely-defined e-nose device types operate based on different and more expensive VOC-detection mechanisms (not utilizing multi-sensor arrays) and include FAIMS, IMR-MS, ion mobility spectrometry, NDIR optical devices and photo-ionization detector (PID). In general, these single-detector, spectrometry-type devices do not depend on the collective output of multiple sensors to generate an overall sensor-array output pattern representative of the complex VOC-analyte gas mixture which are the typical operating methods of traditionally-defined e-nose devices that depend on reference databases of smellprint signatures and sensor pattern-recognition for sample identification.

The types of clinical samples required for e-nose detection and diagnoses of different GI-tract diseases depend on the nature, chemical classes, circulatory-mobility and type of excretion used by the body for elimination of VOC-metabolites associated with particular diseases. This information usually must be determined empirically for each individual disease in order to find the best location(s) where specific disease-associated VOC-metabolites accumulate and can be detected. The ideal excretory sample-type location for acquiring clinical samples for diagnosis of specific diseases is not always intuitive.

The use of diagnostic samples derived from the excretory system such as the lungs, bladder, colon, and skin surface (sweat glands) are most conducive for noninvasive sampling for clinical diagnoses. However, occasionally some diseases require the acquisition of more invasive samples such as blood, swabs from external orifices, esophageal and gastric fluids for effective diagnoses. The need for highly invasive surgical (such as for biopsies), gastroscopic or endoscopic sampling are rarely necessary for very early diagnoses of most GI diseases.

Empirical evidence has indicated that urine samples are most useful for diagnosing bile acid diarrhea, CRC and inflammatory bowel disease. By contrast, colon cancer and irritable bowel syndrome (IBS) may be effectively diagnosed from analysis of exhaled breath or fecal headspace samples, depending on the e-nose device utilized. Most other GI-tract diseases such infectious diarrhea, LOS and NEC may be diagnosed by headspace analysis of fecal VOC-metabolites regardless of e-nose instrument type used. Even though most disease-associated biomarker metabolites form in the parts, organs and compartments of the body where pathogenesis occurs, the relative mobility of disease biomarkers in the circulatory system (which can deliver and concentrate biomarkers into the excretory system) often provide great potential opportunities for disease detections with e-nose devices. Sampling and analysis of headspace volatiles from expelled wastes can come in the form of exhaled breath from the lungs, urine from the kidneys, feces from the colon and sweat glands from the skin. All of these excretory sample types are readily available and conducive for noninvasive collection of clinical samples from patients during routine prophylactic disease-screening examinations and early pre-symptomatic stages of disease development.

### 3.5. Combining E-Nose Analyses with Disease Biomarker Data

The popular development of applications for light-weight portable gas-sensing devices, such as e-noses with VOC-specific sensor arrays, will no doubt gain greater acceptance for routine clinical use as noninvasive early disease-detection methods and procedures are refined and standardized for worldwide use through more extensive clinical testing. World conferences on e-nose clinical uses could help facilitate this standardization process.

The potential for utilizing relatively low-cost e-nose devices to obtain initial diagnostic assessments, through disease VOC-profile signature patterns, followed by confirmations through quick acquisitions of highly disease-specific biomarker data (from the same VOC headspace to support initial diagnoses) provides a powerful tandem diagnostic tool (in combination) for quickly assessing the presence of disease states in a rapid, noninvasive way that could potentially shorten the diagnostic process relative to conventional clinical methods. The detection of disease-specific VOC profiles from e-nose headspace analysis of clinical samples provides a means for noninvasive early detection of disease states in patients, and greatly narrows down diagnostic determinations of probable causes and possible disease etiology based on VOC-signature patterns. Preliminary e-nose diagnoses then may be confirmed by implementing targeted searches for the specific disease biomarker metabolites known to be associated with the most probable diseases identified in initial early disease-screening tests. This diagnostic approach and process is particularly applicable to GI-tract diseases due to the amplification and augmentation of VOC profile changes that occur as pathogenesis alters the microbiome composition in predictable ways for different disease types.

## 4. Future E-Nose Developments for GI-Disease Diagnostics

The discovery of new sensor types and technologies, such as novel electronic-nose devices, offers the potential of additional diagnostic tools for clinical prognoses. Some of the newer e-nose devices utilize detectors with completely different operational mechanisms for detecting VOC metabolites associated with GI-tract diseases. The recent development of an experimental 13-sensor multi-technology e-nose instrument, called the Warwick olfaction system or WOLF e-nose, was developed with the capability to discriminate between CRC and IBS GI-diseases with broadly overlapping symptoms by analysis of lower and higher molecular weight urine headspace volatiles using linear discriminant analysis (LDA) [2]. The WOLF e-nose is composed of a sensor array with eight amperometric electro-chemical (EC) sensors, two nondispersive infrared optical devices and a single photo-ionization detector).

Another unrelated e-nose developed in the same laboratory consists of a nondispersive infrared sensor (IR) optical e-nose, the Warwick Optical Electronic Nose, developed for healthcare applications at POCT facilities [105]. The Warwick Optical E-nose contains four tunable, optical infrared sensors, at the IR range (3.1 to 10.5 µm), based on the detection principle of differential molecular absorption of IR-radiation by sample VOC metabolites known to absorb at specific IR-frequencies. IR adsorption by VOC metabolites results in a decrease in IR-signal as a consequence of specific sensor responses to different VOC gas mixtures present in sample headspace. This new experimental e-nose has been tested with six small molecular weight VOCs, but has not been clinically tested for detection of GI-tract diseases.

The development of new, more sophisticated combination-technology e-nose instruments with some chemical-analysis capabilities, for identifying potential VOC biomarker-metabolites, provides a means for obtaining simultaneous disease-associated volatile signature patterns along with supporting chemical data to qualify the chemical composition of e-nose VOC profiles. Combination-technology devices have been developed in attempts to achieve dual e-nose and chemical simultaneous analyses from the same clinical sample. The Heracles II instrument (Alpha MOS) provides a means for obtaining e-nose signatures (from an extensive collection of available MOS sensors) and GC-data (from dual-column FID detectors) with searchable Kovats-index databases for tentative VOC identifications with analytical standards. A review of some additional older combination-technology e-nose instruments were summarized previously [20].

The detection and identification of bacterial headspace VOCs by GC-MS has been utilized for more than 30 years. However, GC-MS often requires sample collection with VOC-sorbent devices (e.g., SPME) and sometimes compound derivatization, both of which increase analysis time [73]. Recently, SIFT-MS and PTR-MS analysis methods have overcome these limitations for VOC qualification [106,107,108]. Nevertheless, peak fragmentation, an important tool for VOC identification in atmospheric ionization techniques, is not possible with PTR-MS and SIFT-MS. Electrospray plumes potentially may be used to efficiently ionize analytes from the condensed phase [109,110,111,112,113] and gaseous compounds [114,115] using secondary electrospray ionization-mass spectrometry (SESI-MS) [116,117]. These new analytical techniques allow real-time detection and identification of VOCs from clinical samples by MS at detection limits as low as the parts per trillion range [116,118,119,120]. The important emphasis on noninvasive early disease detection and qualitative discriminations of clinical sample types, based on VOC signatures of headspace volatile metabolites, is key to achieving effective early diagnostic determinations and prognoses [121,122]. However, identification of headspace VOCs in clinical samples by metabolomic methods alone does not often lead to effective disease diagnoses without the prior identification of disease-specific VOC biomarker signatures of disease-associated metabolites. Thus, the prior screening of patients for disease using e-nose devices often helps to provide effective preliminary diagnoses, narrowing down the list of likely causes of disease, which can then be more easily confirmed by metabolomic analyses coupled with detection of specific VOC-metabolite disease biomarkers.

## 5. Conclusions

Conventional diagnostic instruments and methods utilized for diagnoses of GI-tract diseases (e.g., colonoscopies, tissue biopsies, microbial culture tests and complex metabolomics analyses) are invasive, expensive, time-consuming and painful to patients [1,3,123]. Considerable recent interest in the potential use of simpler, inexpensive e-nose devices as new diagnostic tools for analyzing VOCs as non-invasive disease biomarkers has stimulated an abundance of new research into replacing existing methods. Several key issues need to be addressed before VOC analysis with e-nose instruments can achieve their full potential as disease detection and monitoring tools for clinical diagnoses. New priority research efforts need to be focused on developing universal standardization of e-nose instruments, sampling protocols, sample transport and storage conditions and analytical methodologies to allow inter-study data comparisons [123,124]. Additional work also is required to identify and expand the list of chemical disease biomarkers known for specific diseases and the unique VOC profile signatures that are indicative of unique complex mixtures of specific biomarker metabolites found in clinical samples which are associated with individual diseases.

New disease-diagnostic concepts were introduced here for the potential use of dual-technology clinical approaches to disease diagnostics that take advantage of the strengths of rapid early prophylactic disease-screening capabilities of e-nose instruments, combined with the quick targeted identification of specific disease biomarkers by metabolomic technologies, if necessary—providing confirmations of preliminary diagnoses to yield rapid, more dependable diagnostic results. Portable electronic-nose devices represent unique EAD technologies that provide new potential analytical tools for simplifying and accelerating POCT clinical diagnostic processes that facilitate more rapid noninvasive early detection of GI-diseases. The result of this diagnostic approach is greater potential for administering earlier more effective and targeted disease-control treatments that improve patient prognoses and significantly shorten recovery times following treatments. This approach involves simultaneous analysis of clinical samples by both instrument types as needed to achieve accuracy in diagnoses.

The examples presented in this review demonstrate recent developments of electronic-nose technologies for POCT and summarize the many ways in which these technologies are beginning to change clinical procedures to reduce time-consuming and expensive laboratory tests and improve the efficiency of patient care and treatments. The development of improved methodologies for achieving accelerated and more accurate clinical disease diagnoses using noninvasive, painless and cheaper procedures support new medical paradigm goals of providing higher quality medical care to patients with faster throughput of diagnostic services. Numerous studies have revealed the efficacy and effective performance of e-nose devices and have shown promising results for the clinical diagnoses of many human diseases, often with the accuracy and sensitivity of slower conventional, established gold-standard diagnostic methods.

## Figures and Tables

**Table 1:** Comparisons of key advantages and disadvantages of metabolomic verses electronic-nose analytical technologies for clinical disease detections and diagnoses.

Technology Types ^1^	Evaluation Criteria	Advantages ^2^	Disadvantages
**Metabolomic**	Analysis costs	Yields more chemistry information and identity of volatile organic compounds (VOCs) in sample	Expensive operating and maintenance costs
	Clinical and field application	Most useful for confirmation of diagnoses by more rapid disease-detection methods; not portable (immobile)	Not suitable due to untimely results and low-sample throughput
	Data analysis	Potentially provides indications of disease mechanisms, host pathways affected, and identity of specific chemical disease biomarkers (more detailed chemical information)	Highly complex, time-consuming, requires sophisticated software and/or models; complex interpretations
	Difficulty level	Provides more details of pathophysiology and metabolic conditions of patient; inter-device data comparisons possible	Labor intensive, requires highly trained operating personnel
	Time requirements to diagnoses	More detailed chemistry information may yield clues for more accurate diagnoses	Slow diagnostic results, not real-time
	Reproducibility	High for clinical samples when prepared with standardized methods & patient histories; low sensor drift over time	Numerous factors may affect volatile organic metabolites (VOMs) and biomarkers identified
**Electronic-nose**	Analysis costs	Relatively inexpensive (low costs); yields simpler collective signature (profile) of all VOC-metabolites present in sample	Individual VOCs not identified (except with combination-technology e-nose instruments)
	Clinical and field application	Highly applicable for clinical use, high sample throughput possible, portable for clinical, patient room or field use	Mobility may be limited by power, weight or space requirements
	Data analysis	Simpler, more straight-forward analyses with easier interpretations of results	Level of sample discrimination is critical
	Difficulty level	Relatively easy to operate and obtain results based on VOC profiles (compared to libraries of reference database)	Inter-device data comparisons (of results) not usually possible
	Time requirements to diagnoses	Relatively rapid preliminary diagnoses; reliability greatly improved with ASRDs, real-time results	Confirmations with other diagnostic data may be required
	Reproducibility	Precision in sensor outputs generally is an asset of e-nose technologies, but results may vary without adequate QA/QC	Sensor drift over time affects reproducibility; sensor poisoning possible

^1^ Technology types for VOC-profile analyses: Metabolomics methods involve the identification and quantification of VOC analytes; electronic-nose methods generally involve analysis of VOCs collectively as a combined profile. ^2^ Data quality-related abbreviations: ASRDs = application specific reference databases; QA/QC = quality assurance/quality control.

**Table 2:** Chemical classes and molecular structures of diverse biomarker metabolites with potential for facilitating the noninvasive early diagnosis of diseases.

Disease ^1^	Pathogen/Cause	Clinical Sample	Biomarker	Chemical Class ^2^	Molecular Structure	Reference
ALS	Neurodegenerative	Blood	butylated hydroxytoluene	Phenol deriv.	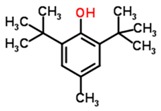	[40]
Bovine TB	*Mycobacterium bovis*	Breath	2,2-dimethyl undecane	Methylated alkane	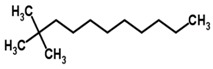	[41]
Cholera	*Vibrio cholerae*	Feces	p-menth-1-en-8-ol	Monoterpene alcohol	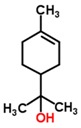	[42]
Cryptosporidiosis	*Cryptosporidium parvum*	Feces	indole	Benzopyrrole	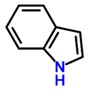	[43]
Endometriosis	Unknown	Endometrial tissue	hypoxanthine	Oxypurine	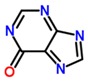	[44]
HNC	Cancer	Urine	2,6-dimethyl-7-octen-2-ol	Terpenoid	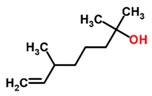	[45]
RCC	Cancer	Urine	2,5,8-trimethyl-1,2,3,4- tetrahydronaphthalene-1-ol	Benzenoid PAH	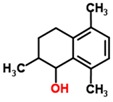	[46]
Stomach ulcer	*Helicobacter pylori*	Breath	2-butanone	Aliphatic ketone	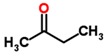	[47]

^1^ Disease name abbreviations: ALS = Amyotrophic Lateral Sclerosis; EM = Endometriosis; HNC = Head and Neck Cancer; RCC = Renal Cell Carcinoma; TB = tuberculosis; ^2^ Abbreviations for chemical classes of biomarkers: PAH = polycyclic aromatic hydrocarbon.

**Table 3:** Biomarker VOC-metabolites discovered with potential application for noninvasive early diagnosis of gastrointestinal (GI) diseases.

GI-Disease ^1^	Pathogen/Cause	N =	Method ^2^	Sample	VOCs	Biomarker VOC-Metabolites (Chemical Class Abbrev.) ^3^	Ref.
Amoebic dysentery	*Cryptosporidium parvum*	50	HIA	Feces	1	Indole (low levels, bpy)	[43,70]
	*Giardia duodenalis*	33	GC-MS	Feces	9	2,2,4,4-tetramethyloctane (ma) 1-propanol (alc) Acetic acid (ca) 2,2,4,6,6-pentamethylheptane (ma) Acetone (ket)	[71]
Bacterial infections (intestinal)	*Campylobacter jejuni*	5	GC-MS	Feces	3+	phenols (bd) indoles (bpy) organic acids (ca)	[42]
		71	GC-MS	Feces	6	Hexanal (ald) (E)-2-octenal (ald) Pyrrole (az) Ethyl ethanoate (es) Methanol (alc) 2-heptanone (ket)	[72]
	*Clostridium difficile*	44	GC-MS	Feces	8	Acetic acid (ca) Butanoic acid (ca) 2-furancarboxaldehyde (ald) 5-methyl-2-furan-carboxaldehyde (ald) Methyl furancarboxylate (fad) 2-hydoxy benzaldehyde (ald) 4-methyl phenol (phed) 2-methoxy phenol (phed)	[42]
	*Escherichia coli*	45	SESI-MS	Culture	3	Acetonitrile (nit) Ethanol (alc) Indole (bpy)	[73]
	*Salmonella typhimurium*	20	GC-MS	Gut	5	Lactose (ds) Melibiose (ds) Raffinose (ts) Fucose (ms) Galactinol (sa)	[74]
	*Shigella flexneri*	80	GC-MS	Culture	2	1-decanol (alc) 1-octanol (alc)	[75]
BAD	Digestive dysfunction	110	FAIMS, GC-MS	Urine	2	2-propanol (alc) Acetamide (amd)	[76]
Cholera	*Vibrio cholerae*	41	GC-MS	Feces	1	2-(4-methyl-3-cyclohexen-1-yl)-2-propanol (mta)	[42]
Coeliac	Gluten sensitive enteropathy	47	FAIMS, GC-MS	Urine	1	1, 3, 5, 7 cyclooctatetraene (cod)	[64]
CRC	Cancer	133	FAIMS	Urine	26	Complex mixture	[63]
CRD	Unknown cause of bowel inflammation	201	IMR-MS	Breath	21	NO_x_ compounds (no) Methane (alk) Ammonia (am) Acetaldehyde (ald)	[77]
IBD	Immune-induced inflammation	117	SIFT-MS	Breath	6	1-octene (ao) 1-decene (alke) (E)-2-nonene (alke)	[61]
IBS	Unknown cause of bowel disorder	323	GC-TOF-MS	Breath	4	1,4-cyclohexadiene (trp) Unidentified VOC Aziridine (azi) n-heptane (alk) n-hexane (alk)	[78]
LOS	Neonatal bacterial infections	35	UPLC-MS	Feces		10,11-dihydro-12R-hydroxy-leukotriene E4 (leu) * Phylloquinone (nqd) * Ascorbic acid (kaad) *	[79]
NEC	Injury-induced intestinal necrosis	65	GC-MS	Feces	4	Absent (present in controls) 2-ethylhexyl acetoate (es) Ethyl decanoate (es) Ethyl dodecanoate (es) Ethyl hexadecanoate (es)	[80]
UC	Abnormal immune response	200	IMR-MS	Breath	21	NO (no) Methane (alk) Ammonia (am) Acetaldehyde (ald)	[77]
VE	Astrovirus, Adenovirus, Norwalk virus	1, 5, 9	GC-MS	Feces	2	Ammonia (am) Ethyl dodecanoate (absent, es)	[42]
	Rotavirus	5	GC-MS	Feces	3	Ethyl dodecanoate (es) Propyl dodecanoate (es) Dodecanoic acid (ca)	[42]

^1^ Disease abbreviations: BAD = Bile acid diarrhea; CRC = Colorectal cancer; CRD = Crohn’s disease; IBD = Inflammatory bowel disease; IBS = Irritable bowel syndrome; ID = Infectious diarrhea; LOS = Late-onset sepsis; NEC = necrotizing enterocolitis; UC = ulcerative colitis, VE = viral enteritis. ^2^ Abbreviations for analytical methods for biomarker identification: FAIMS = field-asymmetric ion Mobility spectrometry; GC-MS = gas chromatography-mass spectrometry; GC-TOF-MS = gas chromatography-time-of-flight mass spectrometry; HIA = hydroxylamine-based indole assay; IMR-MS = ion molecule reaction-mass spectrometry; SESI-MS = secondary electrospray ionization-mass spectrometry; SIFT-MS = selective ion flow tube-mass spectroscopy; UPLC-MS = ultra-performance liquid chromatography-mass spectrometry. ^3^ Chemical class abbreviations of most significant key biomarker VOCs: alc = alcohol; ald = aldehyde; alk = alkane; alke = alkene; am = amine; ao = acyclic olefin; az = azole; azi = aziridine; bd = benzene derivative; bpy = benzopyrrole; ca = carboxylic acid; cod = cyclooctane derivative; ds = disaccharide; es = ester; fad = furoic acid derivative; kaad = ketoaldonic acid derivative; ket = ketone; leu = leukotriene; ma = methylated alkane; ms = monosaccharide; mta = monoterpene alcohol; nqd = naphthoquinone derivative; nit = nitrile; no = nitrogen oxide; phed = phenol derivative; sa = sugar alcohol; trp = terpenoid; ts = trisaccharide; * = chemicals that are nonvolatile organic compounds (NVOMs).

**Table 4:** Recent applications of electronic-nose technologies for the noninvasive early diagnosis of gastrointestinal diseases.

Disease ^1^	Location	Sample	N =	E-Nose Model	Sensor Type/No. ^2^	References
BAD	BAD	Urine	110	Fox 4000	MOS 18	[76]
Cancer	Colon	Breath	26	Experimental	GNP 14	[98]
	Colon	Fecal	157	Cyranose 320	CBPC 32	[5]
CRC/IBD	Colon	Urine	92	WOLF	EC 8, NDIR 2, PID 1	[2]
IBD	Intestine	Urine	62	Owlstone	FAIMS	[99,100]
	Colon	Fecal	83	Cyranose 320	CBPC 32	[101]
IBS	Colon	Fecal	182	Experimental	GC-MOS 1	[102]
	Colon	Breath	234	V&F Airsense	IMR-MS	[77]
ID	Colon	Fecal	100	Experimental	GC-MOS 1	[103]
LOS	Systemic	Fecal	76	Cyranose 320	CBPC 32	[95]
NEC	Colon	Fecal	27	Cyranose 320	CBPC 32	[104]

^1^ Disease abbreviations: BAD = Bile acid diarrhea; CRC = Colorectal cancer; IBD = Inflammatory bowel disease; IBS = Irritable bowel syndrome; ID = Infectious diarrhea; LOS = Late-onset sepsis; NEC = Necrotizing enterocolitis. ^2^ Sensor type abbreviations and number in sensor array: CBPC = carbon black polymer composite; EC = electrochemical sensor; FAIM = field asymmetric ion mobility spectroscopy; GC = gas chromatography; GNP = gold nanoparticles; IMR-MS = ion molecule reaction-mass spectrometry; IMS = ion mobility spectrometry; MOS = metal oxide semiconductor, NDIR = non-dispersive infra-red (optical devices); PID = photo-ionization detector.

## Appendix A

**List of Abbreviations: (Alphabetical)**

ALS	Amyotrophic Lateral Sclerosis
ASRDs	Application-Specific Reference Databases
BAD	Bile Acid Diarrhea
CBPC	Carbon Black Polymer Composite
CC	Colon Cancer
CD	Celiac Disease
CE	Capillary Electrophoresis
CGD	Chronic Gastrointestinal Disease
CNF	Carbon Nanofiber
CP	Conducting Polymer
CRC	Colorectal Cancer
CRD	Crohn’s Disease
EAD	Electronic Aroma Detection
EC	Electrochemical
ED	Ethyl Dodecanoate (biomarker)
E-nose	Electronic-nose
EM	Endometriosis
FAIMS	Field Asymmetric Ion Mobility Spectroscopy
FID	Flame Ionization Detector
GC-MS	Gas Chromatography-Mass Spectrometry
GC-TOF-MS	Gas Chromatography-Time-Of-Flight-Mass Spectrometry
GC/O	GC-Olfactometry
GI	Gastrointestinal
GIRMs	Gastrointestinal Resident Microbes
GNP	Gold Nanoparticle
HIA	Hydroxylamine-based Indole Assay
HNC	Head and Neck Cancer
HS-SPME	Headspace Solid Phase Microextraction
ID	Infectious Diarrhea
IBD	Inflammatory Bowel Disease
IBS	Irritable Bowel Syndrome
IMR-MS	Ion Molecule Reaction-Mass Spectrometry
IR	Infrared
LDA	Linear Discriminant Analysis
LD-PCA	Linear Discriminant Principle Component Analyses
LOS	Late-Onset Sepsis
MIB	2-methylisoborneol (VOC-metabolite)
MOS	Metal Oxide Semiconductors
MS	Mass Spectrometry
NAFLD	Nonalcoholic Fatty Liver Disease
NDIR	Non-Dispersive Infra-red (optical devices)
NEC	Necrotizing Enterocolitis
NMR	Nuclear Magnetic Resonance
NVOMs	Non-Volatile Organic Metabolites
PCA	Principal Component Analysis
PCR	Polymerase Chain Reaction
PID	Photo-Ionization Detector
POCT	Point-Of-Care Testing
PTR-MS	Proton Transfer Reaction Mass Spectrometry
QA/QC	Quality Assurance/Quality Control
QCM	Quartz Crystal Microbalance
RCC	Renal Cell Carcinoma
SAW	Surface Acoustic Wave
SESI-MS	Secondary Electrospray Ionization-Mass Spectrometry
SIFT-MS	Selected Ion Flow Tube-Mass Spectrometry
SPME	Solid Phase Microextraction
TB	Tuberculosis
TCA	Tricarboxylic Acid (pathway)
UC	Ulcerative Colitis
UPLC-MS	Ultra-Performance Liquid Chromatography-Mass Spectrometry
VE	Viral Enteritis
VOCs	Volatile Organic Compounds
VOMs	Volatile Organic Metabolites
